# Flexibility in conceptual combinations: A neural network model of gradable adjective modification

**DOI:** 10.1371/journal.pone.0307775

**Published:** 2024-07-26

**Authors:** Georgia-Ann Carter, Frank Keller, Paul Hoffman

**Affiliations:** 1 Institute for Language, Cognition and Computation, School of Informatics, Edinburgh, United Kingdom; 2 The University of Edinburgh, Edinburgh, United Kingdom; 3 School of Philosophy, Psychology and Language Sciences, Edinburgh, United Kingdom; The University of Lahore, PAKISTAN

## Abstract

Our ability to combine simple constituents into more complex conceptual combinations is a fundamental aspect of cognition. Gradable adjectives (e.g., ‘tall’ and ‘light’) are a critical example of this process, as their meanings vary depending on the noun with which they are combined. For example, a *dark diamond* is less dark than *dark charcoal*. Here, we investigate how a neural network encodes the flexible nature of gradable adjectives in adjective–noun pairs, using the perceptual feature of brightness as a test case. We trained a neural network to predict human brightness ratings for unmodified nouns and adjective–noun pairs and assessed its ability to generalize to untrained combinations (e.g., ‘light paint’ vs. ‘dark paint’). We also explored how this information is encoded. We found that flexible learning of gradable adjectives was possible, with neural networks first making predictions based on the adjective alone, and then modulating these with information from the noun later in learning. We also found that model outputs mimicked the kind of non-additive feature modulation present in human data. Our results have implications for understanding how semantic composition occurs and generate testable predictions for future work.

## 1. Introduction

Conceptual combination refers to the ability to construct complex concepts from simpler constituents. For example, even if you have never encountered combinations such as ‘sand gun’ and ‘robin eagle’, you are able to infer what such concepts may be by relying on the semantics of the constituent words [1,2]. This process is highly dependent on context, with varying outcomes on the semantic representation of the combined phrase. Understanding how conceptual combinations are constructed may be able to help our understanding of conceptual representations in general [2]. Previous theories of conceptual combination from cognitive science have posited two mechanisms: attributive and relational. An attributive process is where an attribute of a word is assigned onto another, such as ‘zebra clam’ to describe a clam with stripes, whereas a relational process concerns the inference of the relationship between two words, such as ‘floor television’ to describe a television standing on the floor [3–5].

What makes adjective–noun combinations such an interesting use-case is their reliance on context in the relation between adjective and noun [6,7], which is the focus of the current study. Adjectives, which are descriptive words that modify the word they attach to [8], are able to modify meaning in multiple ways. Adjective–noun pairs that contain a relative gradable adjective (e.g., ‘tall penguin’) are particularly context-dependent. Solt [9] highlights that these adjectives are only understood in relation to a comparison class. For example, the meaning of adjectives such as ‘tall’ and ‘light’ are understood against the contextual standard for the group of objects that they modify. To explain further, you can use ‘tall’ to describe someone of above average height, and also for a building such as The Shard. Here, the actual height denoted by ‘tall’ is different between the examples as the same adjective can have different effects depending on the noun with which it is paired. As such, the meanings of gradable adjectives are inherently dependent on their context. In general, the adjectival modification of nouns presents an interesting challenge for distributional language models due to the highly variable nature of semantic composition [6,7]. We argue that adjective–noun pairs which include a gradable adjective present an even greater challenge, and that insights from neural network modelling could help us better understand this composition process.

The current study attempts to computationally model this flexibility in conceptual combinations, focusing on adjective–noun pairs. We trained a feedforward neural network to predict brightness ratings for adjective–noun pairs (e.g., ‘light paint’) and tested its ability to generalize to unseen combinations. We presented information about both the unmodified concepts (here represented by a neutral adjective–noun pair condition) and their dark and light combinations. The brightness ratings were taken from Solomon and Thompson-Schill [10], where humans were asked to rate the darkness of concepts for both unmodified nouns and adjective–noun pairs. We compared our model’s performance against the generative models presented by Solomon and Thompson-Schill [10] and present qualitative explorations into how our model performs this task. We found that our model can learn to predict brightness values for adjective–noun pairs and can successfully generalize to unseen adjective–noun combinations, performing at a similar level to Solomon and Thompson-Schill’s Bayesian model, while outperforming simpler additive and multiplicative models. Moreover, we found that our model first learns information about adjective brightness, then begins to combine this additively with knowledge of noun brightness, and only later learns to combine noun and adjective knowledge in the non-additive fashion observed in the human data. Concepts that are more ambiguous with regards to their brightness (e.g., ‘paint’) were also learnt later in training, compared to those that were not (e.g., ‘charcoal’).

To emphasize, the current work is focused on the question of *how* models encode this information, rather than concerns about performance, as this can provide novel insights and generate further hypotheses about the process of semantic composition. It has recently been suggested that neural networks are a promising method for capturing both the systematic and idiosyncratic aspects of language, due to the lack of constraints imposed on the internal representations used when mapping inputs to outputs [11]. Thus, it is possible that these types of models would be useful in modelling the flexibility of gradable adjectives, which combine systematic constraints with idiosyncratic item-related biases to construct a meaningful interpretation.

## 2. Related work

Much of the computational work on semantic composition has implemented vector- and matrix-based compositional functions to represent combined concepts [12–14]. Hartung et al. [13] aimed to model adjectival attribute meanings using word embeddings. Attribute selection is the task of predicting the hidden attribute meaning that is expressed by an adjective–noun combination; for example, the difference between understanding that ‘hot summer’ relates to the temperature of the combined concept, whilst a ‘hot debate’ relates to the passion surrounding the topic. By making use of a dataset with attribute annotations, they found that weighted combinations of adjective and noun embeddings could accurately predict the attribute described by a phrase, outperforming predictions from either the adjective or noun alone [13]. While their findings on how meaning is represented in adjective–noun pairs is of interest, the investigations in Hartung et al. [13] only predict which attribute can be assigned to the adjectival modifier (e.g., weight, brightness, speed), rather than the magnitude of the modifier’s influence. Thus, the question of how to build flexibility into computational representations of gradable adjective–noun pairings remains.

Shwartz and Dagan [15] identified six tasks associated with compositional phenomena and tested how well a range of word embeddings could accurately reflect the lexical composition process. Overall, they found that contextualized word embeddings performed better at the tasks, compared to static embeddings. However, while they exhibit similar performance to humans at recognizing meaning shifts, performance was much lower for tasks that required a representation of implicit meaning. This highlights the difficulty distributional models have in representing the meanings of phrases, especially those with contextually-dependent interpretations [6,7,15].

**Fig 1:**
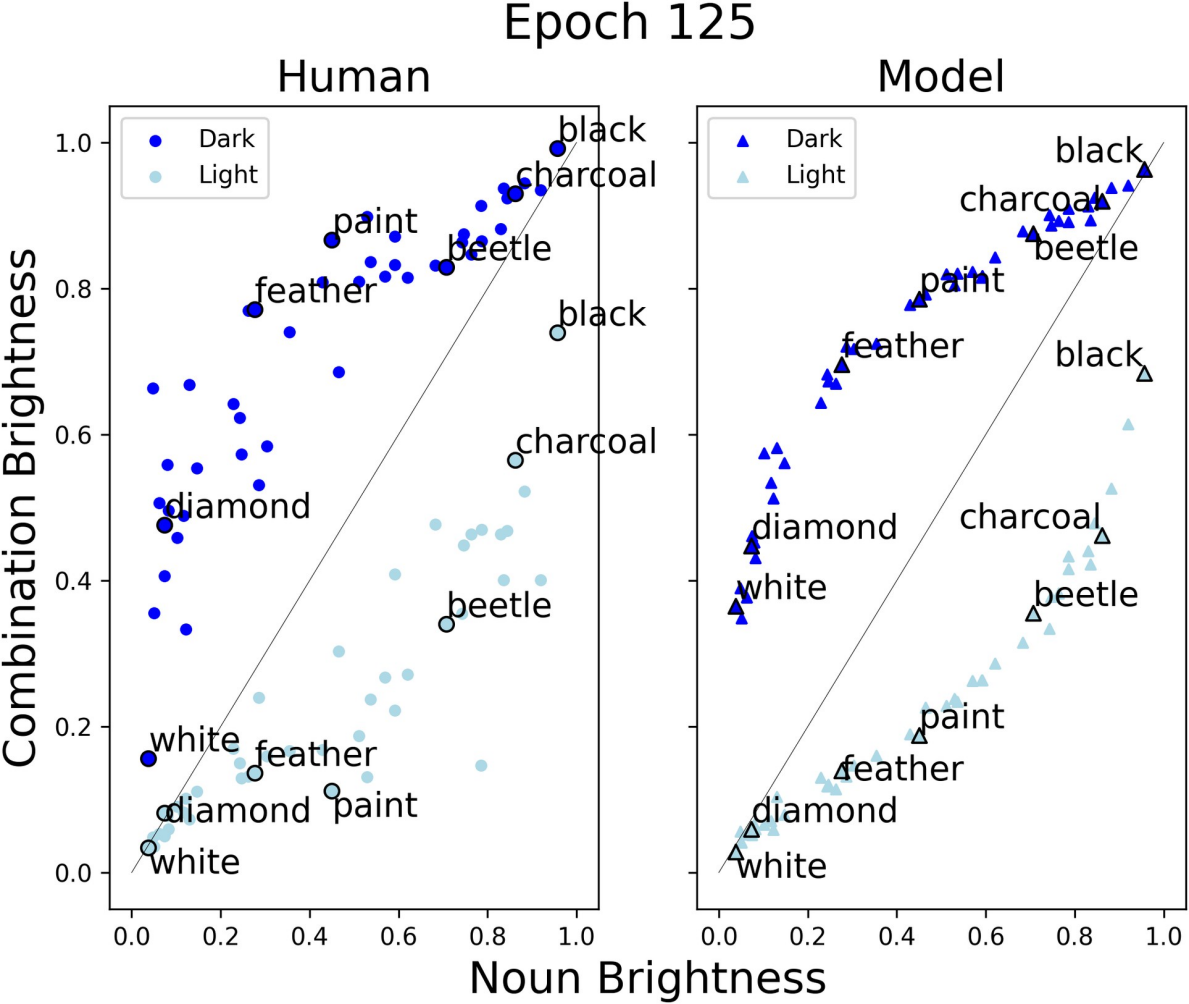
Human ratings and model predictions of combined brightness. Human ratings of combination brightness for all concepts (left); model predictions of combination brightness for held-out concepts after training (right).

Solomon and Thompson-Schill [10] have recently attempted to model flexibility in conceptual combinations. They used a three-pronged approach, incorporating behavioural, computational and neuroimaging methods to explore conceptual structure and the neural regions that support the flexible use of features. The authors focused on the level of perceptual brightness conveyed by adjective–noun pairs. They introduced a construct, feature uncertainty, which reflects the entropy associated with a concept’s brightness [16]. In their behavioural experiments, human participants rated the brightness of 45 modified and unmodified concepts on a scale from 0 (light) to 50 (dark) (see ‘Human’ plot in Fig 1). They found that brightness ratings were influenced by both the adjective and noun. For example, the ratings for ‘light feather’ were lighter than those for either ‘dark feather’ or ‘light charcoal’. It was also apparent that the degree to which the adjective modulated brightness was not constant across nouns. For example, some of the concepts had large differences between their light and dark modified forms (e.g., ‘paint’), whereas for other concepts, this difference was much smaller (e.g., ‘white’). The authors found that the flexible modulation of brightness across concepts correlated with their construct of feature uncertainty: the degree of adjectival modulation was greatest for objects of moderate brightness (e.g., ‘paint’, ‘slippers’; which were assumed to have the greatest feature uncertainty), and smallest for objects with more extreme values of brightness (e.g., ‘snow’, ‘charcoal’). As such, their data suggests a predictable, but non-additive relationship between the expected brightness of an adjective–noun pair and the brightness of its adjective and noun constituents.

The authors also implemented a number of generative models for brightness prediction. They incorporated two baselines, where the predicted brightness of the combination was just the brightness of either the noun or adjective, respectively. They also included an additive model, which predicted combination brightness through a weighted sum of adjective and noun brightness; a multiplicative model, which predicted combination brightness through a scaled product of adjective brightness and noun brightness; and a Bayesian model, which generated predictions through a product of Gaussian brightness distributions for the adjective and noun, fit on the response frequencies from the behavioural judgement task. They found that the Bayesian model significantly outperformed the other models. As the Bayesian model was the only model to incorporate information on feature uncertainty (i.e., the variability in brightness ratings for each object), the authors argued that feature uncertainty was critical for capturing the patterns of feature modulation in the human judgements [10].

In the present study, we investigated how a simple neural network learns to predict the brightness of adjective–noun concepts. The network was trained on a subset of Solomon and Thompson-Schill’s [10] adjective–noun brightness ratings and tested on its ability to predict brightness for unseen, novel combinations of adjectives and nouns. This work represents an advance on previous work in two ways. First, existing models provide accounts of how adjective and noun information combines in a mature semantic system but are largely silent on how this ability is acquired. As neural networks learn to perform tasks incrementally through training, they provide an opportunity to investigate how representations emerge and what developmental stages are involved [17,18]. Second, unlike the Bayesian model proposed by Solomon and Thompson-Schill, our simulations included no notion of feature uncertainty. This allowed us to test whether the construct of feature uncertainty is necessary to account for non-linear effects of adjectival modification.

## 3. Methods

### 3.1 Dataset

The dataset from Solomon and Thompson-Schill [10] consists of averaged human ratings from a behavioural experiment, where human raters were asked to rate the brightness of unmodified nouns (e.g., ‘coffee’) and modified adjective–noun pairs (e.g., ‘light coffee’ vs ‘dark coffee’) for 45 concepts. The original dataset from Solomon and Thompson-Schill [10] can be accessed here: https://osf.io/7uwn9/. Two separate groups of participants (*n* = 100; *n* = 199) rated the brightness of the unmodified nouns and the brightness of the adjective–noun combinations. The brightness ratings were on a scale from 0 to 50, with 0 representing light and 50 representing dark.

We used the averaged ratings of the brightness of the unmodified concepts and the averaged ratings of the brightness of the combined concepts (for example, the concept ‘black’ had an unmodified rating of 47.83, while ‘dark black’ had a rating of 49.61 and ‘light black’ a rating of 37). We transformed these ratings to a scale from 0 to 1, with 1 now representing the dark end of the spectrum. To standardize our inputs to our model, we appended a brightness-agnostic adjective (‘neutral’) to the unmodified concepts. Therefore, we had three versions of each concept: dark, light and neutral, resulting in 135 items in total. To generate our model inputs, we created a one-hot encoding of both the noun and the adjective, and then combined these to form a representation of the adjective–noun pairs.

### 3.2 Model

We implemented a feedforward neural network architecture in PyTorch [19]. The network consisted of three layers, with one hidden layer. The input layer consisted of 48 units, representing the 45 nouns and 3 adjectives. The hidden layer had 30 units, while the output layer consisted of 1 unit, which represented the model’s brightness prediction. Between the linear layers, we included a Rectified Linear Unit (ReLU) activation function [20], while we used a Sigmoid activation function between the hidden and output layers in order to transform the model prediction between 0 and 1, and thus be comparable to our scaled brightness ratings.

We set a range of hyperparameters, with some values optimized through grid search (see Section 3.3), and others taken from a study with a similar goal of representing flexibility in semantic concepts [21]. As such, our model had a *bias* = -2, *momentum* = 0.9, and *weight decay* = 10^−6^.

### 3.3 Training

Due to the limited size of our dataset, we implemented k-fold cross validation (*k* = 10) in order to maximize the utility of our data [22–24]. We chose *k* = 10 as it has been widely used across the machine learning literature [24–26]. We split our dataset into train and test sets, with approximately 122 items in train and 13 items in test. We ensured that the nouns present in the adjective–noun pairs in the test set were also present in a different combination in the train set. For example, if ‘dark charcoal’ was a test item for one of our folds, then we confirmed that the train set contained at least one ‘charcoal’ item, such as ‘neutral charcoal’. We fed the input items to the model in batches, with a batch size of 14. We performed hyperparameter optimization using nested k-fold cross validation (*n* = 3), such that our training set was further split into three sets, with one of these sets used as a validation set. We implemented grid search, whereby we optimized on learning rate, the number of hidden units and the number of epochs for training [27]. We evaluated our grid search using the negative mean squared error (MSE), which resulted in optimal parameters of *learning rate* = 0.3, *number of hidden units =* 30 and *train time in epochs* = 125. In our final models, training ran for 125 epochs, with our model weights optimized through stochastic gradient descent [28].

### 3.4 Evaluation

To evaluate our model’s predictions on the unseen adjective–noun pairs, we used mean-squared error, comparing the model brightness predictions for the unseen adjective–noun pairs against the ground-truth, i.e., averaged brightness ratings from human participants, and R^2^. As such, during training, the model acquires knowledge about the typical brightness of a range of objects and is shown how the two adjectives (‘dark/light’) modulate brightness for some, but not all, of these objects. It is then tested on the combinations that were not provided during training. Thus, we tested the model’s ability to acquire knowledge about how dark/light adjectives modulate the expected brightness of objects, situated along the brightness spectrum, and then to generalize this knowledge to novel adjective–noun combinations.

## 4. Results

We trained 10 models initialized with different random weights. Each model was trained for 10 iterations using k-fold cross-validation. All results below are averaged over the 10 models and only include performance on unseen adjective–noun combinations.

### 4.1 Model performance

In Fig 1, we plot the model predictions for the held-out combined concepts against the human ratings, after training for the full number of epochs. These are separated by adjective, with annotated examples taken from Solomon and Thompson-Schill [10]. The brightness of the unmodified concept is plotted on the x-axis, against the predictions of combination brightness on the y-axis. Here, a value of 0 refers to the lightest possible object, while 1 refers to the darkest items. The grey line across the plots indicates the alignment of the combination brightness with the brightness of the unmodified concept. The model performed comparatively well in predicting the combination brightness of concepts after the full training procedure. Further, the model captured the three main features of the human data: (1) that the brightness of the adjective–noun pair is influenced both by the adjective and the noun, (2) that the degree to which the adjective modulates the brightness varies across nouns and (3) that the largest modulations occur for nouns of moderate brightness.

**Fig 2:**
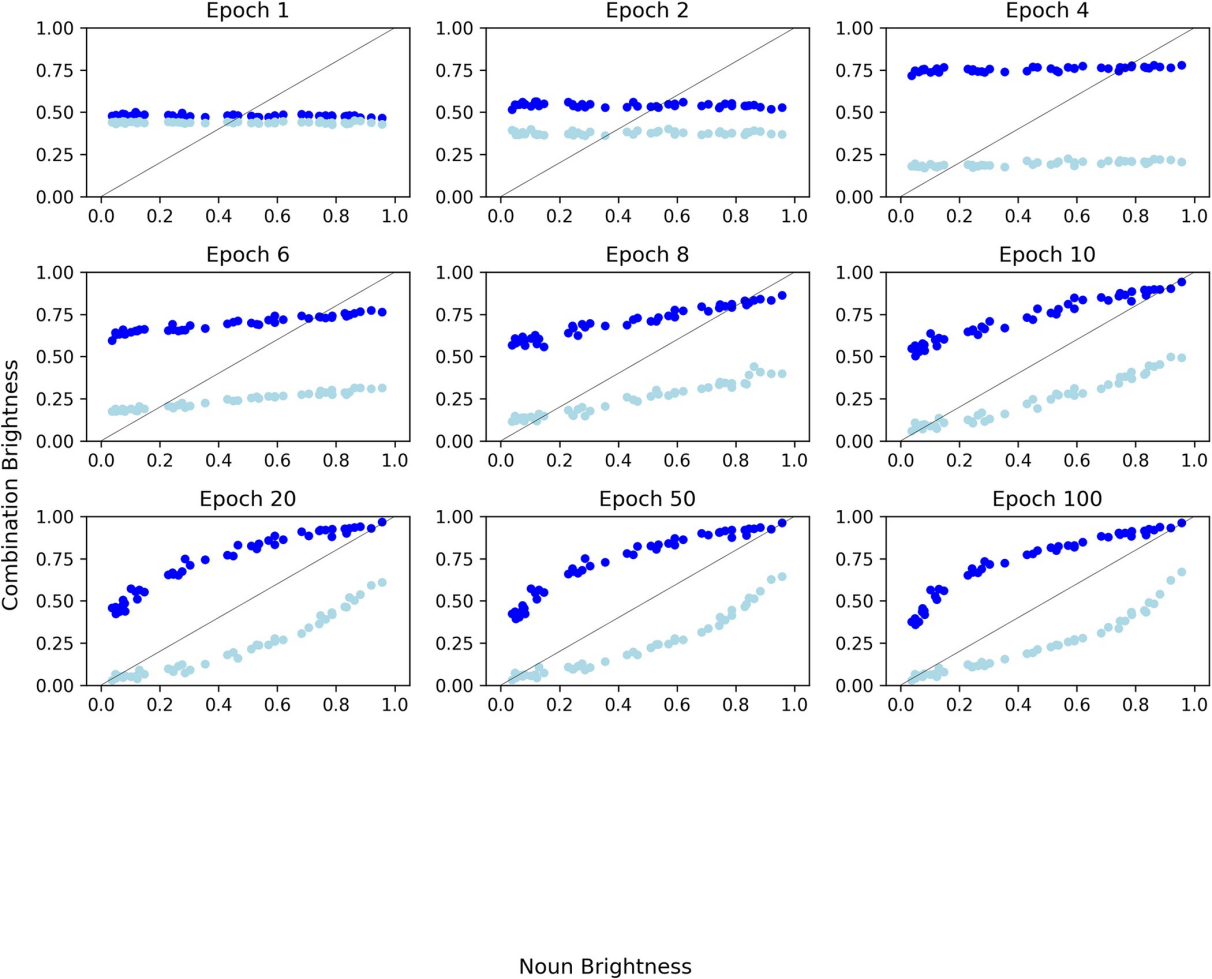
Model predictions of combination brightness during a subset of epochs.

To investigate how the model evolved during training, we plot model predictions across a subset of epochs during the training procedure (see Fig 2). The model passes through a series of developmental stages. The model begins to cluster the combined concepts early on during training by making brightness predictions based on the adjective alone. The subplot of Epoch 4 highlights this clearly, with the ‘dark’ items depicted in dark blue, and the ‘light’ items depicted in light blue. The model is correctly predicting the difference between ‘dark’ and ‘light’ items but is insensitive to the noun brightness. However, by Epoch 10, the model acquires knowledge of noun brightness in order to assign a more accurate prediction (i.e., combination brightness begins to be influenced by noun brightness). Here, the model’s predictions resemble the additive model from Solomon and Thompson-Schill [10], in that the model is sensitive to the brightness of both the noun and the adjective, but the adjective modulates each noun’s brightness to the same extent. This modulation becomes more flexible and noun-dependent in the later epochs, as demonstrated by the eventual non-linear curves in the Epoch 100 plot. Here, it appears that the model is gradually refining its predictions as it learns that the adjectives can have variable influences on different nouns, for example, a greater influence for concepts that fall in the centre of the brightness spectrum. Solomon and Thompson-Schill [10] suggest that this feature of the human data is due to a greater amount of uncertainty for moderate-brightness nouns. However, there was no uncertainty in the inputs to our model—each adjective–noun combination was associated with a single, fixed brightness value. This suggests that the non-additive modulation patterns in the human data can be explained without appealing to feature uncertainty.

### 4.2 Model comparison

In order to ascertain how our neural network performed in comparison to the models presented in Solomon and Thompson-Schill [10], we replicated their models using the data provided by the original authors. We also transformed the neural network predictions back to the original brightness scale (0 = light, 50 = dark) for comparability. We then evaluated the models’ performances using mean squared error (MSE), R^2^, and the standard deviation, and compared these across all models. Results can be found in Table 1, with results from our model depicted in bold. We also performed statistical analyses on the squared errors of the combinatorial models. A one-way ANOVA (analysis of variance) demonstrated that overall MSEs differed across the four models (*F*_(3, 176)_ = 19.22, *p* <0.01). Pairwise comparisons revealed that the Bayesian and neural network models did not differ significantly in performance (*t*_*(*44)_ = 0.08, *p* = 0.94). The neural network did significantly outperform both the additive and multiplicative models, however (*t*_(44)_ = 2.24, *p* = 0.03; *t*_*(*44)_ = 5.13, *p* < 0.01).

**Table 1:** Model comparisons. Bold indicates our implementation; all other implementations are from Solomon and Thompson-Schill (2020). % change indicates the percentage difference in MSE between our neural network and all other implementations.

Model	MSE	SD	R^2^	% change
Adjective	258.63	25.0	0.00	93.7
Noun	207.30	14.88	0.09	92.1
Additive	29.57	17.76	0.87	44.5
Multiplicative	80.24	20.47	0.65	79.6
Bayesian	16.87	14.85	0.93	2.7
**Neural network**	**16.41**	**15.13**	**0.93**	

### 4.3 Learning trajectories

To understand the mechanisms that supported learning, we ran qualitative explorations into the model’s performance. We first outline our investigations into the learning trajectories of the annotated examples across epochs. After, we discuss the activations of the hidden representations and present a cluster-based analysis using t-SNE (t-distributed stochastic neighbour embedding) [29].

**Fig 3:**
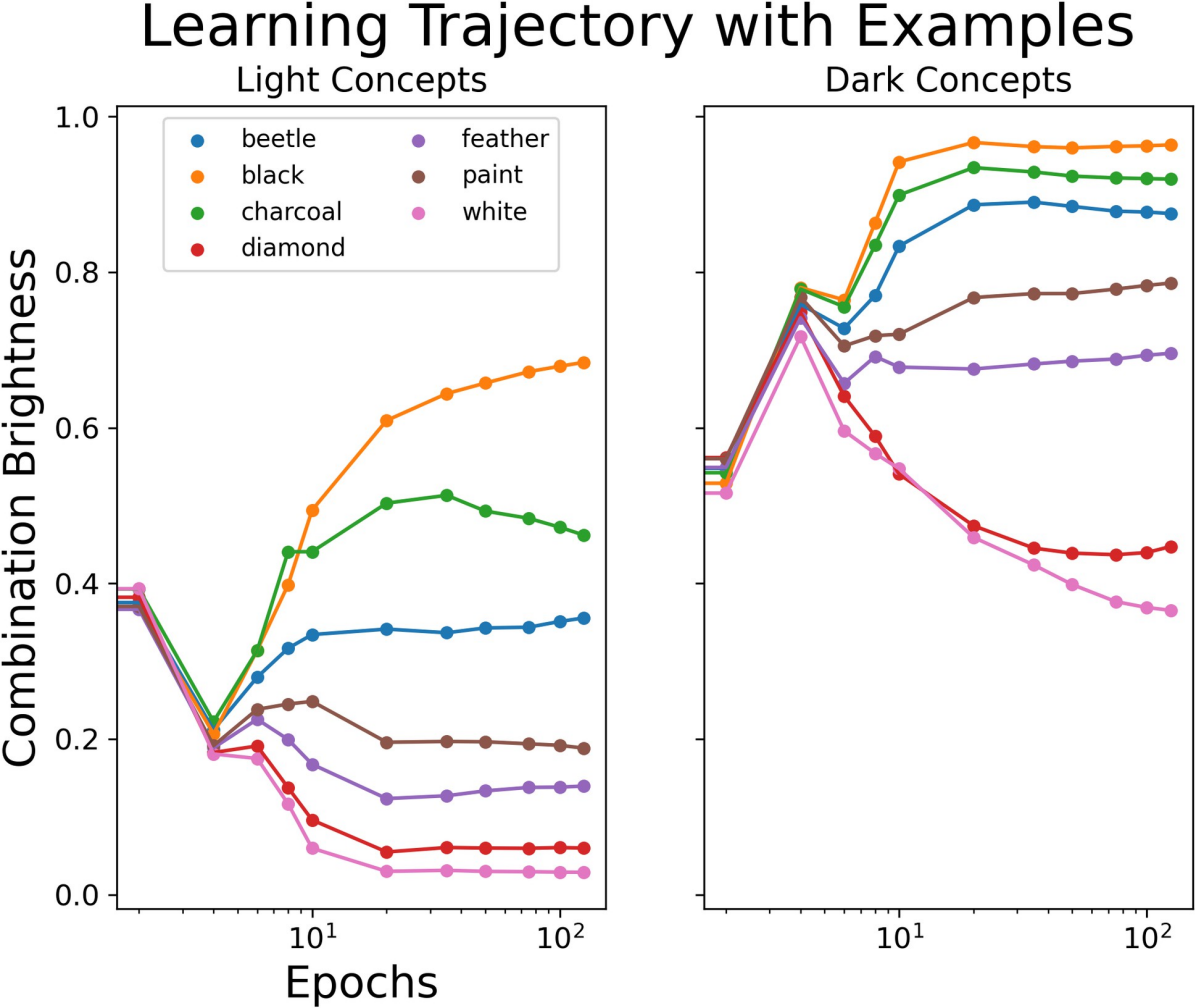
Model predictions for annotated examples over training. Model predictions of combination brightness across epochs (on a log-scale) for selected concepts, separated by adjective (light items on the left plot; dark items on the right plot).

To understand how our model performs on selected cases, we used the annotated examples shown in Fig 1. These contain concepts across the range of brightness ratings. Fig 3 depicts the model predictions of the combined concepts, separated by adjective. We plot these predictions across epochs on a log-scale to better demonstrate the distinction in predictions between the earlier and later epochs. Fig 3 shows that the predictions for the combined concepts become distinguishable by noun only later during training. This again highlights the clustering of combined brightness by adjective that dominates the model’s initial predictions.

**Fig 4:**
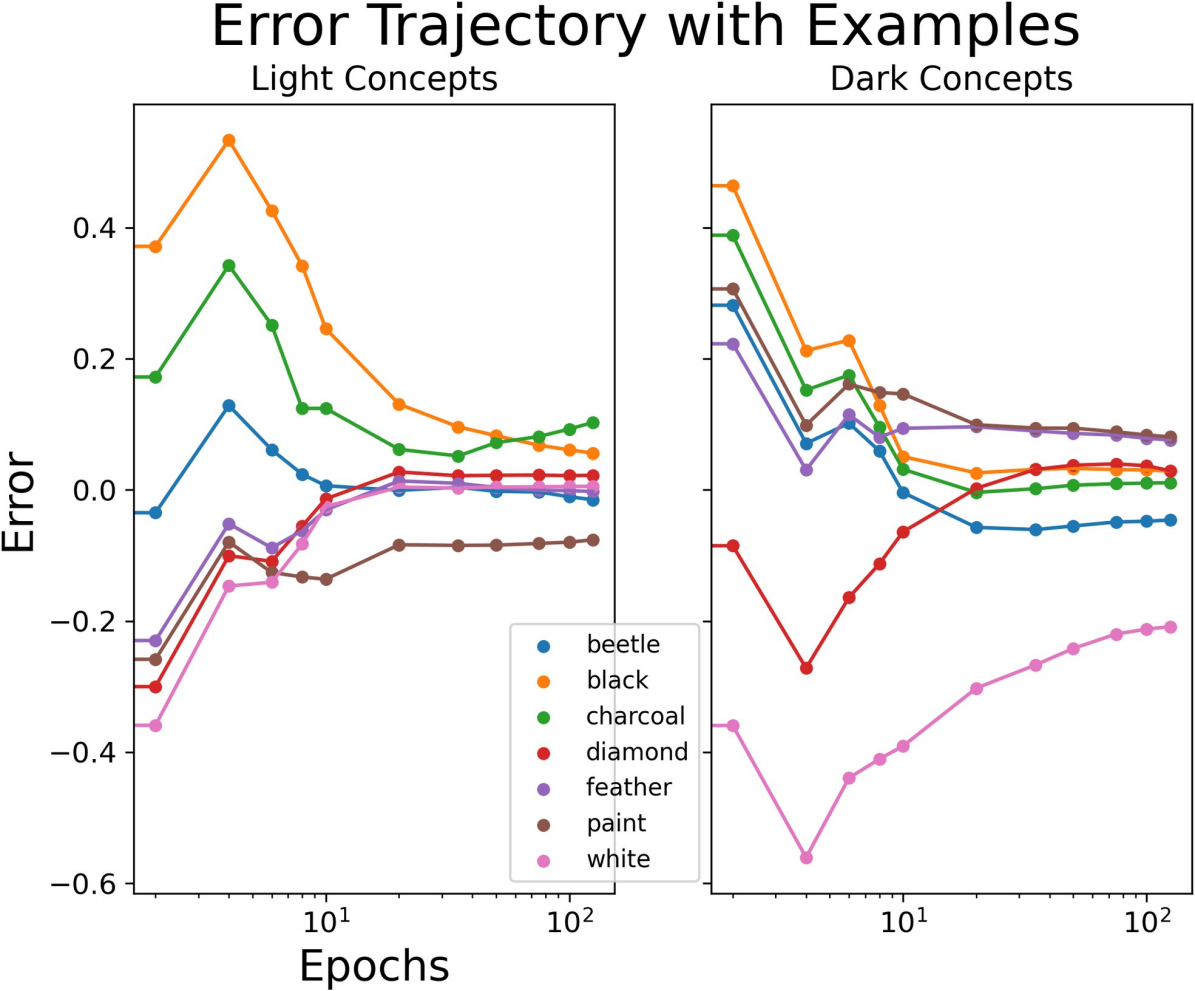
Error between model predictions and ground-truth during training. Prediction error of combined brightness predictions against true values across epochs (log-scaled) for selected concepts, separated by adjective (light items on the left plot; dark items on the right plot).

We also investigated the error in model predictions for these annotated examples across all epochs (see Fig 4). Here, we define error as the numerical difference between the model predictions and the true combined brightness value. As such, negative values indicate that the model’s predictions were darker than the true combined brightness value (i.e., closer to 1), whereas positive values represent model predictions that were lighter than the true combined brightness value (i.e., closer to 0). The large peaks in the error values are another indication of the model’s predictions first assigning similar values to combinations with the same adjective. For example, the high error peak for ‘light black’ (see the orange peak in the left plot), compared with the high error peak for ‘dark white’ (see the pink peak in the right plot). This shows that the model is slowest to learn appropriate brightness predictions for concepts where the adjective and noun have contradictory brightness associations.

### 4.4 Hidden representation analysis

**Fig 5:**
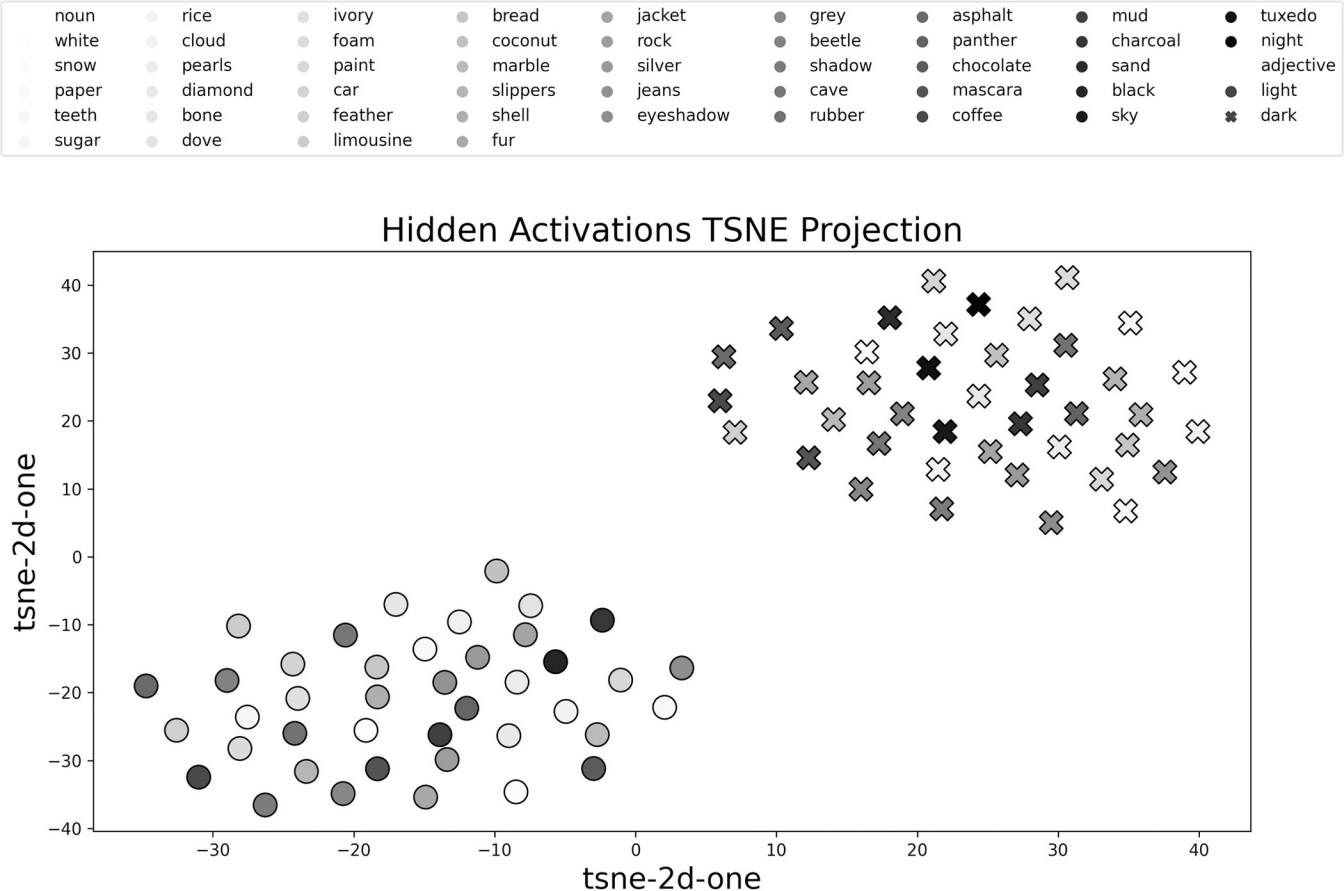
Hidden activations after 2D-TSNE reduction. Adjectives depicted by marker shape (circles represent light items; crosses represent dark items); nouns depicted through greyscale colour (ordered by unmodified brightness).

Finally, we analysed the hidden representations acquired by our neural network. We extracted the hidden activations for each item after training (epoch 125). We then performed dimensionality reduction using t-SNE to reduce the hidden activations from 30 dimensions to 2 dimensions. We ran this procedure for each of our folds to ensure that our output was consistent. Here, we only depict a representative figure from one of our 10 folds. In Fig 5, we can observe that the hidden activations of our items form two clusters based on the adjective, with light items represented by the circles, and dark items represented by the crosses. The darkness of the points refers to the unmodified noun brightness. In none of the investigations of the hidden activations did we find clusters based on the noun. This reinforces our previous findings that adjective identities are the dominant organizing principle for the model’s representations.

## 5. Discussion

In this study, we investigated how a neural network learns to encode the flexible nature of semantic composition with relative gradable adjectives. We trained a small neural network to predict brightness ratings of a range of concepts using both unmodified nouns and adjective–noun pairs. When tested with novel adjective–noun combinations, our model implementation performed as well as the Bayesian model presented in Solomon and Thompson-Schill [10]. While both models exhibit similar MSE and R^2^, the Bayesian implementation is fit with a richer dataset (i.e., the distribution of ratings across individual participants), whereas our neural network achieves similar performance using only the mean ratings. As such, we argue that our neural network demonstrates an improvement over the Bayesian model due to the requirement for less training data. In addition, our neural network does not make use of the novel construct of feature uncertainty, which suggests that data on the uncertainty of a property is not needed to predict the influence of gradable adjectives on adjective–noun pairs.

Furthermore, the nature of neural networks allowed us to investigate exactly how the model predictions developed across training. Our investigations into the learning trajectories of specific examples revealed that the network first clustered the items by adjective. Later in training, the influence of the noun’s brightness plays a role, with model predictions assuming an additive nature whereby the adjective modulates each noun to the same extent. Towards the end of training, model predictions finally converge on a non-additive mapping, whereby the adjectives exert differential modulation of the combination brightness, depending on the nouns they are paired with. One question that emerges is whether children acquire knowledge about gradable adjectives in the same way. Previous research into the acquisition of gradable adjectives has demonstrated that children as young as 4 years old are able to interpret adjectives in a way that is sensitive to the statistics of the object class they are applied to [30,31]. However, there is little evidence on earlier stages of acquisition, so it is currently unknown whether children first develop adjective-based representations before this noun-specific information is incorporated. It was also found that children demonstrated an asymmetry in their mastery of compositional semantics with regards to positive and negative terms (e.g., better mastery of ‘tall’, compared with ‘short’) (see [23]). We did not find this asymmetry within our neural network, as predictions for both light and dark items appeared to develop similarly across epochs (i.e., first assign a blanket adjective prediction, then nuance by noun). It is possible this is because we did not provide any information as to the valence of the items. In other words, the model is not aware of which adjective corresponds to a positive or negative term in the real world. It is also possible that the age of acquisition (AoA) of positive and negative terms influences this asymmetry. One suggestion for further research would be to replicate this asymmetry in the acquisition of compositional semantics to better understand the mechanisms surrounding the influence of context on combined concepts. For example, a possible AoA influence could be introduced through focusing training on lighter items during earlier epochs.

A key question that arises from the findings of the current study is whether this approach extends to other conceptual properties. The use of brightness as our property of interest has some caveats, in the sense that it is strongly perceptually grounded. It has been demonstrated that perceptually salient cues are particularly important in the grounding of cognition [32]. It is possible that less perceptually grounded and concrete properties, such as ‘expensive’, are less amenable to this approach, especially considering the greater individual variability in rating more abstract concepts [33]. The extension of this approach to other properties is, therefore, an interesting direction for future research. For example, further investigations with both different conceptual features of interest and adjectival types could assess whether the organizing patterns we observe here are general features of adjective–noun combination.

One limitation of the current approach is the simultaneous presentation of the adjective and noun representations to the model. As such, we were not able to investigate the impact of sequential presentation on compositional semantics. The use of sequential models would allow us to further investigate the mechanisms that support compositional semantics in spoken language. With more complex sequential models, such as a model trained to predict the noun following a presented adjective as well as its expected brightness, future research could also focus on the interplay between language and embodied perceptual predictions. This would enable further exploration into the nature of statistical influence on the acquisition of compositional semantics, and thus, how the preceding context supplies comprehenders (whether human or artificial) with prior expectations that shape the semantic interpretation of a noun.

## 6. Conclusions

This study has demonstrated that neural networks are able to flexibly learn mappings of gradable adjectives onto unmodified nominal concepts. Our neural network implementation quantitatively performs similar to previous implementations, and also provides a window into the acquisition of this type of compositional semantic structures. We found that early predictions were organized by adjective representations, with influence of the noun appearing later. We also found that the model is slowest to learn appropriate brightness predictions for concepts where the adjective and noun have contradictory brightness associations. These findings provide further insight into the mechanisms by which conceptual combination may occur and allow for more targeted hypothesis generation for future studies into the phenomena.
